# Neem (*Azadirachta indica* L*.*) leaf extract deteriorates oocyte quality by inducing ROS-mediated apoptosis in mammals

**DOI:** 10.1186/2193-1801-3-464

**Published:** 2014-08-26

**Authors:** Shail K Chaube, Tulsidas G Shrivastav, Meenakshi Tiwari, Shilpa Prasad, Anima Tripathi, Ajai K Pandey

**Affiliations:** Cell Physiology Laboratory, Biochemistry Unit, Department of Zoology, Banaras Hindu University, Varanasi, 221005 India; Department of Reproductive Biomedicine, National Institute of Health and Family Welfare, Baba Gang Nath Marg, Munirka, New Delhi, 110067 India; Department of Kayachikitsa, Faculty of Ayurveda, Banaras Hindu University, Varanasi, 221005 India

**Keywords:** Aqueous neem leaf extract, Reactive oxygen species, Granulosa cell, DNA fragmentation, Mitochondria-mediated oocyte apoptosis

## Abstract

Neem (*Azadirachta indica* L*.*) leaf has been widely used in ayurvedic system of medicine for fertility regulation for a long time. The molecular mechanism by which neem leaf regulates female fertility remains poorly understood. Animal studies suggest that aqueous neem leaf extract (NLE) induces reactive oxygen species (ROS) - mediated granulosa cell apoptosis. Granulosa cell apoptosis deprives oocytes from nutrients, survival factors and cell cycle proteins required for the achievement of meiotic competency of follicular oocytes prior to ovulation. Under this situation, follicular oocyte becomes more susceptible towards apoptosis after ovulation. The increased level of hydrogen peroxide (H_2_O_2_) inside the follicular fluid results in the transfer of H_2_O_2_ from follicular fluid to the oocyte. The increased level of H_2_O_2_ induces p53 activation and over expression of Bax protein that modulates mitochondrial membrane potential and trigger cytochrome c release. The increased cytosolic cytochrome c level induces caspase-9 and caspase-3 activities that trigger destruction of structural and specific proteins leading to DNA fragmentation and thereby oocyte apoptosis. Based on these animal studies, we propose that NLE induces generation of ROS and mitochondria-mediated apoptosis both in granulosa cells as well as in follicular oocyte. The induction of apoptosis deteriorates oocyte quality and thereby limits reproductive outcome in mammals.

## Introduction

Neem plant (*Azadirachta indica* L*.*) has been considered as one of the most important medicinal plants worldwide. The medicinal utility of this plant are listed in ancient documents ‘Charak-Samhita’ and ‘Susruta-Samhita’ that are considered as the foundation of the Indian system of natural treatment, Ayurveda (Girish and Shankara Bhat 2008). It is considered as ‘Sarvaroga nivarini’ that means the curer of all ailments (Subapriya and Nagini 2005). The various parts of neem plant are used for the treatment of several diseases in ayurvedic system of medicine worldwide. The aqueous extract of neem bark has therapeutic potential for controlling gastric hypersecretion and gastroduodenal ulcer (Bandyopadhyay et al. 2004), while neem leaf extract has been used to reduce oral infections, plaque index and bacterial count (Pai et al. 2004a, [b]).

### Medicinal properties of Neem Leaf

Medicinal properties of neem leaf have already been reviewed (Subapriya and Nagini 2005). Neem leaf extract (NLE) exhibit anti-inflammatory, anti-hyperglycemic, anti-ulcer, immunomodulatory, antiviral, anti-fungal, anti-bacterial, nematicidal, anti-malarial, insecticidal, anti-mutagenic and anti-oxidant properties (Biswas et al. 2002; Sharma et al. 2003; Wandscheer et al. 2004; Udeinya et al. 2004; Siddiqui et al. 2004; Subapriya and Nagini 2005; Sithisaran et al. 2005). The Anti-helminthic activity of NLE has also been reported in ruminents (Al-Rofaai et al. 2012). One of the bioactive fractions of neem leaf (nimbolide) has anti-cancer property (Harish Kumar et al. 2010). The apoptosis inducing ability of NLE has been investigated in cancer cells (Dharmalingam et al. 2011). The NLE induces cytoplasmic granulation and deteriorates oocyte quality suggesting its potential use for female fertility regulation in brown dog ticks (Denardi et al. 2010). The anti-fertility properties of neem extracts have been reported in several mammalian species (Mukherjee et al. 1999; Subapriya and Nagini 2005).

Several studies have been carried out to find the role of neem products in male fertility regulation. The neem bark ethereal extract induces reversible changes in reproductive system of male rats and resulting into male infertility (Raji et al. 2003). Neem oil treatment reduces tubular diameter, inhibits spermatogenesis in rat (Shaikh et al. 2009) and sperm motility in mice (Yin et al. 2004).The ethanolic NLE induces abnormal head morphology and reduces mean sperm count in murine (Khan and Awasthi 2003). The aqueous NLE inhibits motility and viability of human spermatozoa under in vitro culture conditions (Khillare and Shrivastav 2003). Neem oil has been used as herbal vaginal contraceptive in human (Sharma et al. 1996).

The role of NLE on female fertility regulation remains poorly understood. Few studies suggest that neem oil reduces number of developing follicles in the rat ovary and induces degeneration of oocyte in vitro (Juncia and Williams 1993; Dhaliwal et al. 1999; Roop et al. 2005). The neem oil inhibits implantation in rats and bonnet monkeys and acts as a reversible contraceptive (Upadhyay et al. 1990, 1994; Garg et al. 1998). However, neem oil has unpleasant sharp odour and thus becomes unpalatable. Hence, aqueous NLE could be used as an alternative herbal medicine for fertility regulation in mammals including human since it does not possess an unpleasant characteristic sharp odour of neem (Selvamurthy 1997).

### Generation of ROS in ovary and oocyte quality

Ovary is a dynamic organ and generates excess amount of reactive oxygen species (ROS) during final stages of folliculogenesis and ovulation, while their effects are neutralized by active enzymatic antioxidant system (Agarwal et al. 2005; Fujii et al. 2005; Sugino 2005). A moderate increase of ROS under physiological range could be beneficial for meiotic resumption from diplotene arrest in mammalian oocytes cultured in vitro (Agarwal et al. 2005; Chaube et al. 2005; Pandey et al. 2010). However, overproduction of ROS in ovary or depletion of enzymatic antioxidant system may results in oxidative stress (Agarwal et al. 2005). The increased oxidative stress level can reduce oocyte quality by inducing apoptosis, fertilization and pregnancy rates in mouse as well as in human (Tamura et al. 2008).

### NLE triggers ROS-mediated granulosa cell and oocyte apoptosis

The mechanism by which NLE has a direct access at the level of mammalian ovary and oocyte remains unclear. Animal studies suggest that NLE reduces ovary weight, ovulation rate (Gbotolorun et al. 2008), inhibits folliculogenesis and antrum formation in follicles (Mukherjee et al. 1999; Dhaliwal et al. 1999; Roop et al. 2005). Studies from our laboratory suggest that NLE triggers apoptosis in preovulatory follicles, reduces number of granulosa cells encircling oocyte, induces dispersion of granulosa cells and oocyte apoptosis in majority of ovulated cumulus oocytes complexes (COCs) (Tripathi et al. 2012, 2013).

The aqueous NLE decreases catalase activity that results in the accumulation of hydrogen peroxide (H_2_O_2_) in rat ovary (Chaube et al. 2006; Tripathi et al. 2013). The increased level of H_2_O_2_ acts as an upstream signal to induce p53 and Bax protein expression (Chaube et al. 2006; Tripathi et al. 2013). The overexpression of Bax protein modulates mitochondrial membrane potential and increases cytochrome c release in cell cytoplasm (Chaube et al. 2006; Tripathi et al. 2013). A rise in cytochrome c concentration induces DNA fragmentation and thereby granulosa cell apoptosis (Chaube et al. 2006; Tripathi et al. 2013).

The granulosa cell apoptosis results in the disruption of gap junctions between encircling somatic cell and oocyte inside the follicular microenvironment. Reduced cell-cell communication deprives oocyte from nutrients, maturation-enabling factors and survival factors inside the preovulatory follicle and reduces oocyte quality by inducing susceptibility towards apoptosis (Tripathi et al. 2013). The granulosa cell apoptosis also reduces estradiol 17-β level required for development and maturation of oocytes in the ovary. The hypo-estrogenic condition inside the follicular microenvironment may affect development and maturation of oocytes and trigger generation of ROS and mitochondria-caspase-mediated pathway (Chaube et al. 2006; Tripathi et al. 2012, 2013). Hence, the follicular oocyte is unable to achieve meiotic competency and become susceptible towards apoptosis after ovulation (Chaube et al. 2006; Tripathi et al. 2012, 2013).

Quercetin is one of the major bioactive flavonoids and constitute to 6 to 48% (w/w) of NLE (Subapriya and Nagini 2005; Sithisarn and Gritsanapan 2008). Quercetin inhibits antioxidant systems (thioredoxin or glutathione) that increases ROS level and thereby apoptosis (Pelicano et al. 2004; Kuo et al. 2007; Jeong et al. 2009). Studies from our laboratory suggest that quercetin induces cell shrinkage, membrane leakage, cytoplasmic granulation and cytoplasmic fragmentation in treated oocytes prior to degeneration (Chaube et al. 2006; Tripathi et al. 2012, 2013). These morphological apoptotic changes were associated with the increased H_2_O_2_ level, overexpression of Bax protein, caspase-3 activation and DNA fragmentation (Chaube et al. 2006; Tripathi et al. 2012, 2013). Based on these studies, we propose that NLE and its bioactive ingredient such as quercetin induce ROS-mediated granulosa cell apoptosis followed by oocyte apoptosis. The NLE-induced oocyte apoptosis deteriorates oocyte quality that finally reduces reproductive outcome (Chaube et al. 2006; Tripathi et al. 2012, 2013).

### Future prospects

NLE is ecologically friendly, safe and biologically active botanical substance that induces apoptosis in follicular granulosa cells as well as in oocyte that deteriorates oocyte quality after ovulation. The apoptosis inducing property of NLE makes it a potential candidate for the development of reversible herbal contraceptive for the control of female fertility in mammals. The apoptotic inducing property of NLE may also be used to control the rodent population in the field that damage almost 30% crops worldwide. NLE could be mixed in common edible food of rodents and pellets can be made. These pellets can be kept in rodent prone areas for their easy access. This neem product formulation could replace various harmful rodenticides used frequently by the farmers that can affect human health directly or indirectly by passing on to next trophic level.

## Conclusions

**Figure 1:**
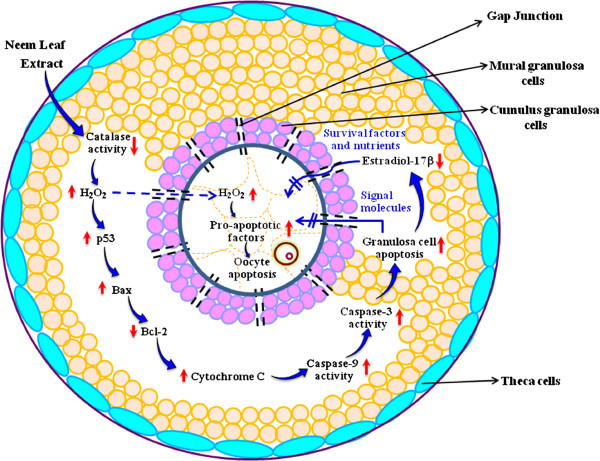
**A schematic diagram showing the possible mechanism of NLE action at the level of follicle in mammalian ovary.** NLE inhibits catalase activity and induces the accumulation of H_2_O_2_ inside the follicular microenvironment of the ovary. The increased H_2_O_2_ level induces granulosa cell apoptosis through mitochondria-caspase-mediated pathway. Granulosa cell apoptosis leads to disruption of gap junction, interruption in the transfer of signal molecules from cumulus cells to the oocyte and reduction of estradiol 17-β level further deprive oocyte from nutrients and survival factors. The H_2_O_2_ also enters directly in the oocyte and trigger oocytes apoptosis. Under this condition, pro-apoptotic factors are upregulated and oocyte becomes susceptible towards apoptosis. NLE induced apoptosis leads to the deterioration in the oocyte quality and limit reproductive outcome in mammals.

NLE reduces catalase activity and increases the accumulation of H_2_O_2_ inside follicular microenvironment of the mammalian ovary. The increased H_2_O_2_ level induces granulosa cell apoptosis through mitochondria-caspase-mediated pathway. Granulosa cell apoptosis reduces estradiol 17-β level in ovary and induces disruption of gap junctions between granulosa cells and oocytes. Hence, oocyte is deprived of cell signaling molecules, nutrients and survival factors required for the achievement of meiotic competency of oocyte just prior to ovulation. Under this condition, pro-apoptotic factors are upregulated thus making oocytes susceptible towards apoptosis after ovulation (Figure 1). Thus, NLE-induced oocyte apoptosis deteriorates oocyte quality and limits reproductive outcome in mammals.

## Authors’ information

Author SKC is the Principal Investigator of a project entitled “Studies on neem (*Azadirachta indica* L*.*) leaf extract-induced egg apoptosis in rat” funded by Department of Science and Technology, Ministry of Science and Technology, Government of India, New Delhi.

## Abbreviations

COCs: Cumulus oocytes complexes
H_2_O_2_: Hydrogen peroxide
NLE: Neem leaf extract
ROS: Reactive oxygen species.

## References

[CR1] Agarwal A, Gupta S, Sharma R (2005). Oxidative stress and its implications in female infertility-a clinician’s perspective. Reprod Biomed Online.

[CR2] Al-Rofaai A, Rahman WA, Sulaiman SF, Yahaya ZS (2012). In vitro activity of neem (*Azadirachta indica*) and cassava (*Manihot esculenta*) on three pre-parasitic stages of susceptible and resistant strains of *Teladorsagia* (Ostertagia) *circumcincta*. Vet Parasitol.

[CR3] Bandyopadhyay U, Biswas K, Sengupta A, Moitra P, Dutta P, Sarkar D (2004). Clinical studies on the effect of neem (*Azadirachta indica*) bark extract on gastric secretion and gastroduodenal ulcer. Life Sci.

[CR4] Biswas K, Chattopadhyay I, Banerjee RK, Bandyopadhyay U (2002). Biological activities and medicinal properties of neem (*Azadirachta indica*). Curr Sci.

[CR5] Chaube SK, Prasad PV, Thakur SC, Srivastava TG (2005). Hydrogen peroxide modulates meiotic cell cycle and induces morphological features characteristics of apoptosis in rat oocytes cultured in vitro. Apoptosis.

[CR6] Chaube SK, Prasad PV, Khillare B, Shrivastav TG (2006). Extract of *Azadirachta indica* (Neem) leaf induces apoptosis in rat oocytes cultured in vitro. Fertil Steril.

[CR7] Denardi SE, Bechara GH, Oliveira PR, Camargo-Mathias MI (2010). *Azadirachta indica A. Juss* (neem) induced morphological changes on oocytes of *Rhipicephalus sanguineus* (Latreille, 1806) (Acari: Ixodidae) tick females. Exp Parasitol.

[CR8] Dhaliwal PK, Roop JK, Guraya SS (1999). Effect of neem-seed oil on the quantitative aspects of follicular development in cyclic female rats. Indian J Ecol.

[CR9] Dharmalingam N, Gunadharini PE, Ramachandran A, Kalimuthu S, Jagadeesan A (2011). Induction of apoptosis and inhibition of PI3K/Akt pathway in PC-3 and LNCaP prostate cancer cells by ethanolic neem leaf extract. J Ethanopharmacol.

[CR10] Fujii J, Iuchi Y, Okada F (2005). Fundamental role of reactive oxygen species and protective mechanism in the female reproductive systems. Reprod Biol Endocrinol.

[CR11] Garg S, Taluja V, Talwar GP, Upadhyay SN (1998). Immunocontraceptive activity guided fractionation and characterization of active constituents of neem (*Azadirachta indica*) seed extracts. J Ethnopharmacol.

[CR12] Gbotolorun SC, Osinubi AA, Noronha CC, Okanlawon AO (2008). Antifertility potential of Neem flower extract on adult female Sprague–Dawley rats. Afr Health Sci.

[CR13] Girish K, Shankara Bhat S (2008). Neem – a green treasure. e J Biol.

[CR14] Harish Kumar G, Priyadarsini RV, Vinothini G, Vidjaya LP, Nagini S (2010). The neem limonoids azadirachtin and nimbolide inhibit cell proliferation and induce apoptosis in an animal model of oral oncogenesis. Invest New Drugs.

[CR15] Jeong JH, An JY, Kwon YT, Rhee JG, Lee YJ (2009). Effects of low dose quercetin: cancer cell-specific inhibition of cell cycle progression. J Cell Biochem.

[CR16] Juncia SC, Williams RS (1993). Mouse sperm-egg interaction in vitro in the presence of neem oil. Life Sci.

[CR17] Khan PK, Awasthi KS (2003). Cytogenetic toxicity of neem. Food Chem Toxicol.

[CR18] Khillare B, Shrivastav TG (2003). Spermicidal activity of *Azadirachta indica* (neem) leaf extract. Contraception.

[CR19] Kuo PL, Chen CY, Hsu YL (2007). Isoobtusilactone A induces cell cycle arrest and apoptosis through reactive oxygen species/apoptosis signal-regulating kinase 1 signaling pathway in human breast cancer cells. Cancer Res.

[CR20] Mukherjee S, Garg S, Talwar GP (1999). Early post implantation contraceptive effects of a purified fraction of neem (*Azadirachta indica*) seeds, given orally in rats: possible mechanisms involved. J Ethnopharmacol.

[CR21] Pai MR, Acharya LD, Udupa N (2004). Evaluation of antiplaque activity of *Azadirachta indica* leaf extract gel-a six-week clinical study. J Ethanopharmacol.

[CR22] Pai MR, Acharya LD, Udupa N (2004). The effect of two different dental gels and a mouthwash on plaque and gingival scores: a 6-week clinical study. Int Dent J.

[CR23] Pandey AN, Tripathi A, Premkumar KV, Shrivastav TG, Chaube SK (2010). Reactive oxygen and nitrogen species during meiotic resumption from diplotene arrest in Mammalian Oocytes. J Cell Biochem.

[CR24] Pelicano H, Carney D, Huang P (2004). ROS stress in cancer cells and therapeutic implications. Drug Resist Update.

[CR25] Raji Y, Udoh US, Mewoyeka OO, Ononye FC, Bolarinwa AF (2003). Implication of reproductive endocrine malfunction in male infertility efficacy of *Azadirachta indica* extract in rats. Afr J Med Sci.

[CR26] Roop JK, Dhaliwal PK, Guraya SS (2005). Extracts of *Azadirachta indica* and *Melia azedarach* seeds inhibit follicullogenesis in albino rats. Braz J Med Biol Res.

[CR27] Selvamurthy W (1997). Herbal contraceptive in the works. Science.

[CR28] Shaikh MA, Naqvi SNH, Chaudhary MZ (2009). Effect of neem oil on the structure and function of the mature male albino rat testes. Braz J Morphol Sci.

[CR29] Sharma SK, SaiRam M, Ilavazhagan G, Devendra K, Shiavaji SS, Selvamurthi W (1996). Mechanism of action of NIM-76: a novel vaginal contraceptive from neem oil. Contraception.

[CR30] Sharma V, Walia S, Kumar J, Nair MG, Parmar BS (2003). An efficient method for the purification and characterization of nematicidal azadirectins A, B, and H, using MPLC and ESIMS. J Agric Food Chem.

[CR31] Siddiqui BS, Rasheed M, Ilyas F, Gulzar T, Tariq RM, Naqvi SN (2004). Analysis of insecticidal *Azadirachta indica. A. Juss.* fractions. Z Naturforsch C.

[CR32] Sithisaran P, Supabphol R, Gritsanapan W (2005). Antioxidant activity of Siamese neem tree (VP1209). J Ethanopharmacol.

[CR33] Sithisarn P, Gritsanapan W (2008). Variability of antioxidative quercetin content in Siamese neem tree leaves in Thailand by TLC densitometry. Acta Hort (ISHS).

[CR34] Subapriya R, Nagini S (2005). Medicinal properties of neem leaves: a review. Curr Med Chem Anti-Cancer Agents.

[CR35] Sugino N (2005). Reactive oxygen species in ovarian physiology. Reprod Med Biol.

[CR36] Tamura H, Takasaki A, Miwa I, Taniguchi K, Maekawa R, Asada H, Taketani T, Matsuoka A, Yamagata Y, Shimamura K, Morioka H, Ishikawa H, Reiter RJ, Sugino N (2008). Oxidative stress impairs oocyte quality and melatonin protects oocytes from free radical damage and improves fertilization rate. J Pineal Res.

[CR37] Tripathi A, Shrivastav TG, Chaube SK (2012). Aqueous extract of *Azadirachta indica* (neem) leaf induces generation of reactive oxygen species and mitochondria-mediated apoptosis in rat oocytes. J Assist Reprod Genet.

[CR38] Tripathi A, Shrivastav TG, Chaube SK (2013). An increase of granulosa cell apoptosis mediates aqueous neem (*Azadirachta indica*) leaf extract induced oocyte apoptosis in rat. Int J Appl Basic Med Res.

[CR39] Udeinya IJ, Mbah AU, Chijioke CP, Shu EN (2004). An antimalarial extract from neem leaves is antiretroviral. Trans R Soc Trop Med Hyg.

[CR40] Upadhyay SN, Kaushic C, Talwar GP (1990). Antifertility effects of neem (*Azadirachta indica*) oil by single intrauterine administration: a novel method for contraception. Proc Biol Sci.

[CR41] Upadhyay SN, Dhawan S, Sharma MG, Talwar GP (1994). Long-term contraceptive effects of intrauterine neem treatment (IUNT) in bonnet monkeys: an alternate to intrauterine contraceptive devices (IUCD). Contraception.

[CR42] Wandscheer CB, Duque JE, da Silva MA, Fukuyama Y, Wohlke JL, Adelmann J, Fontana JD (2004). Larvicidal action of ethanolic extracts from fruit endocarps of *Melia azedarach* and *Azadirachta indica* against the dengue mosquito *Aedes aegypti*. Toxicon.

[CR43] Yin Z, Jia R, Gao P, Gao R, Jiang D, Liu K, Liu S (2004). Preparation of contraceptive pill microcapsule and its antifertility effect. Sheng Wu Yi Xue Gong Cheng Xue Za Zhi.

